# CD4^+^ T cells from COVID-19 mRNA vaccine recipients recognize a conserved epitope present in diverse coronaviruses

**DOI:** 10.1172/JCI156083

**Published:** 2022-03-01

**Authors:** Bezawit A. Woldemeskel, Arbor G. Dykema, Caroline C. Garliss, Saphira Cherfils, Kellie N. Smith, Joel N. Blankson

**Affiliations:** 1 Department of Medicine, and; 2 Bloomberg-Kimmel Institute for Cancer Immunotherapy, Johns Hopkins Medicine, Baltimore, Maryland, USA.; 3 Sidney Kimmel Comprehensive Cancer Center, Johns Hopkins University, Baltimore, Maryland, USA.; 4 Hunter College, CUNY, New York, New York, USA.

**Keywords:** COVID-19, Adaptive immunity

## Abstract

Recent studies have shown that vaccinated individuals harbor T cells that can cross-recognize SARS-CoV-2 and endemic human common cold coronaviruses. However, it is still unknown whether CD4^+^ T cells from vaccinated individuals recognize peptides from bat coronaviruses that may have the potential of causing future pandemics. In this study, we identified a SARS-CoV-2 spike protein epitope (S_815-827_) that is conserved in coronaviruses from different genera and subgenera, including SARS-CoV, MERS-CoV, multiple bat coronaviruses, and a feline coronavirus. Our results showed that S_815-827_ was recognized by 42% of vaccinated participants in our study who received the Pfizer-BioNTech (BNT162b2) or Moderna (mRNA-1273) COVID-19 vaccines. Using T cell expansion and T cell receptor sequencing assays, we demonstrated that S_815-827_-reactive CD4^+^ T cells from the majority of responders cross-recognized homologous peptides from at least 6 other diverse coronaviruses. Our results support the hypothesis that the current mRNA vaccines elicit T cell responses that can cross-recognize bat coronaviruses and thus might induce some protection against potential zoonotic outbreaks. Furthermore, our data provide important insights that inform the development of T cell–based pan-coronavirus vaccine strategies.

## Introduction

The COVID-19 pandemic has caused about 318 million infections and more than 5.5 million deaths since its emergence in Hubei province, China, in December 2019 (1). SARS-CoV-2, the virus that causes COVID-19, may have originated in bats (2–5). In the past 20 years, 2 additional highly pathogenic and transmissible coronavirus outbreaks with possible bat origins have occurred: SARS-CoV, which emerged in 2003, and MERS-CoV, which emerged in 2012 (3). Surveillance studies have shown that bats are reservoirs for SARS-related and other genetically diverse coronaviruses (6). Zoonotic infections from bat-borne coronaviruses thus pose a major threat to humans, and the development of vaccines that can elicit robust, cross-reactive immunity across many coronaviruses is essential to protect against future pandemics (7).

Multiple vaccine candidates with high efficacy and immunogenicity against the original SARS-CoV-2 strain have recently been developed and administered worldwide (8–11). The COVID-19 mRNA vaccines generate strong T cell responses against the original SARS-CoV-2 strain (12) and variants of concern (13, 14). Robust T cell responses are associated with less severe COVID-19 infection (15), and T cell immunity has been shown to be protective against SARS-CoV-2, SARS-CoV, and MERS-CoV infections in animal models (16–18). In the past year, multiple groups have described the presence of cross-reactive T cells that can cross-recognize SARS-CoV-2 and endemic human common cold coronaviruses (HCoVs) (19–28). Additionally, the Pfizer-BioNTech (BNT162b2) and Moderna (mRNA-1273) vaccines have been shown to enhance HCoV-NL63–specific T cell responses after vaccination (14), suggesting enhanced vaccine-mediated immunity against this common cold coronavirus. T cell cross-reactivity is likely a result of sequence homology between SARS-CoV-2 and HCoVs. Computational studies have identified a highly conserved region within the fusion peptide domain of SARS-CoV-2 spike protein (29, 30). We have recently shown that some mRNA vaccine recipients target a peptide within this conserved region (S_813-829_) (14). Furthermore, Loyal et al. have recently shown that more than 90% of COVID-19–vaccinated individuals in their cohort harbor T cells that recognize a peptide located within the fusion peptide domain (S_816-830_) and that these T cells are cross-reactive (31). Because S_813-829_ is highly conserved in diverse coronaviruses, we hypothesized that vaccinated individuals could recognize this conserved epitope from bat coronaviruses not known to infect humans.

To test our hypothesis, we analyzed T cell responses against the conserved SARS-CoV-2 spike epitope S_815-827_ in individuals who received 2 doses of COVID-19 mRNA vaccines. Our findings suggest that mRNA-vaccinated individuals have T cell responses that can cross-recognize multiple bat coronaviruses not currently known to infect humans. Our study will have implications for the development of T cell–oriented pan coronavirus vaccines that could protect against future zoonotic coronavirus outbreaks.

## Results

**Figure 1:**
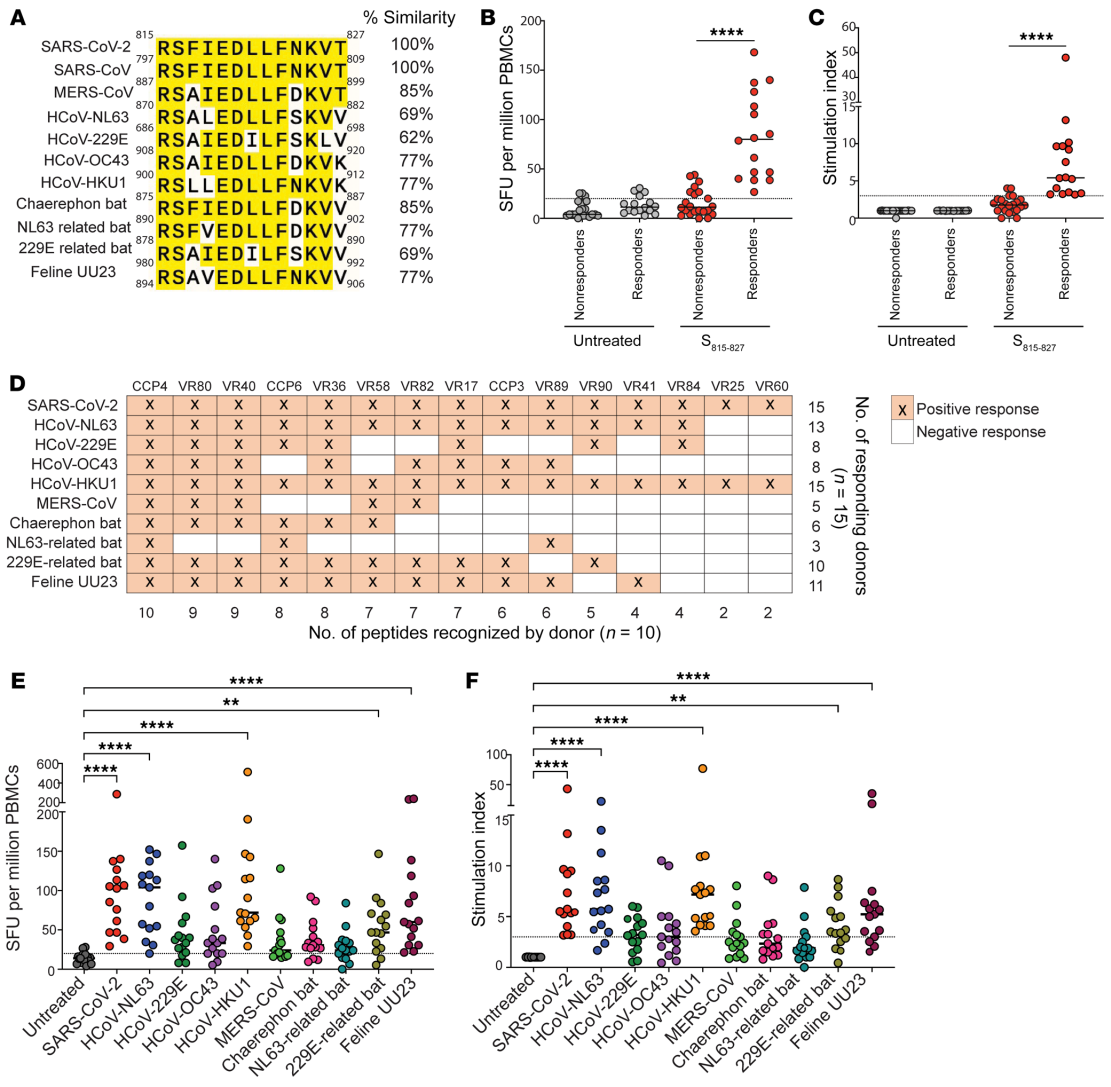
Some individuals vaccinated with COVID-19 mRNA vaccines have CD4^+^ T cells that recognize the conserved SARS-CoV-2 epitope S_815-827_ and homologous peptides from diverse coronaviruses. Sequence alignment for coronavirus peptides used in this study are shown (**A**). CD8^+^ T cell–depleted PBMCs were isolated from 38 vaccinated individuals, and an IFN-γ ELISpot assay was done in triplicate with S_815-827_ or untreated control. Mean of replicates was used to plot spot-forming units (SFUs) (**B**) and stimulation index (SI) (**C**). Responders (*n* = 16) and nonresponders (*n* = 22) were above our cutoff of SFU ≥ 20, and SI ≥ 3. S_815-827_ responders (*n* = 15) were further assessed for CD4^+^ T cell responses to homologous coronavirus peptides with IFN-γ ELISpot (**D**–**F**). Positive CD4^+^ T cell responses based on our cutoff for each individual donor and corresponding peptide are shown in orange (**D**). SFU and SI for donors are also shown (**E** and **F**, respectively). Mann-Whitney test (**B** and **C**) and Friedman’s test with Dunn’s multiple-comparison test (**E** and **F**) were used for statistical comparisons. *P* values below 0.05 were considered statistically significant. **P = 0.0021, ****P < 0.0001.

Cross-reactive T cells are likely a result of sequence homology between SARS-CoV-2 and endemic HCoVs (29, 30). The SARS-CoV-2 spike peptide S_815-827_ is found within the fusion peptide domain of the SARS-CoV-2 spike and is highly conserved in alpha and betacoronaviruses (29) (Figure 1A and Table 1). Additionally, the SARS-CoV-2 S_815-827_ peptide sequence is identical in some coronaviruses found in the *Sarbecovirus* subgenus (Supplemental Table 1; supplemental material available online with this article; https://doi.org/10.1172/JCI156083DS1).

In this study, we looked at T cell responses in vaccinated individuals to S_815-827_ and to homologous peptides from coronaviruses isolated from diverse hosts, including humans, bats, and felines (Table 1). We previously identified the 17-mer peptide S_813-829_ to be targeted by CD4^+^ T cells in some COVID-19 mRNA vaccine recipients (14). In this study, we synthesized 15-mer and 13-mer truncated peptides and performed IFN-γ enzyme-linked immunosorbent spot (ELISpot) assays in 3 vaccine recipients in order to determine the minimal peptide recognized by reactive T cells. We identified a 13mer sequence S_815-827_ (RSFIEDLLFNKVT) to be comparably recognized (Supplemental Figure 1A), and we proceeded to use this peptide for further experiments.

We next asked whether S_815-827_ is recognized by the majority of COVID-19–vaccinated individuals. To test this, we isolated CD8^+^ T cell–depleted PBMCs from 38 individuals vaccinated with Pfizer-BioNTech (BNT162b2) or Moderna (mRNA-1273) vaccines and performed IFN-γ ELISpot assays. All 38 individuals tested positive for antibodies to all 4 HCoVs by commercial ELISA kits, indicating prior exposure to these viruses. We found that 16 out of 38 (42%) of our donors (termed hereafter as responders) had robust T cell responses to S_815-827_ and were above our cutoff of spot-forming unit (SFU) greater than or equal to 20 and stimulation index (SI) greater than or equal to 3 (Figure 1, B and C). For 3 donors for whom we had cryopreserved prevaccination samples, we performed IFN-γ ELISpot assays to determine whether responses to S_815-827_ existed prior to COVID-19 vaccinations. None of the donors tested had responses to S_815-827_ prior to vaccinations (Supplemental Figure 1, B and C), indicating that at least in these donors, responses to S_815-827_ were induced or expanded by vaccination. All responders had the HLA allele DPA1*01:03, and most had the predicted combined HLA binding allele DPA1*01:03/DPB1*04:01, suggesting that this might be a restricting allele for S_815-827_ (Table 2). We have previously shown that lymphoblastoid cell lines with DPA1*01:03/DPB1*04:01 are capable of presenting the related peptide S_813-829_ (19).

Given that S_815-827_ is a highly conserved epitope, we hypothesized that COVID-19–vaccinated individuals will have T cells that recognize homologous peptides from diverse coronaviruses with zoonotic potential. To test this hypothesis, we isolated CD8^+^ T cell–depleted PBMCs from individuals who responded to S_815-827_ and performed an IFN-γ ELISpot assay using homologous peptides from 9 coronaviruses, including HCoVs, MERS-CoV, bat coronaviruses, and a feline coronavirus (listed in Table 1). We found that all donors recognized at least 1 other coronavirus peptide, and 8 out of 15 donors recognized peptides from at least 6 out of the 9 other coronaviruses tested (Figure 1D). The coronaviruses most robustly recognized were common cold coronaviruses (HCoV-NL63, HCoV-HKU1), 229E-related bat coronavirus, and feline UU23 coronavirus (Figure 1, E and F).

Previous studies have shown that HCoV and SARS-CoV-2 cross-reactive CD4^+^ T cells have lower functional avidity than SARS-CoV-2 monoreactive T cells (19, 26). Given that there could be functional avidity differences in T cells responding to S_815-827_ and corresponding homologous peptides, we performed a peptide titration in 3 donors using the IFN-y ELISpot assay. Overall, we did not observe major differences in functional avidity to S_815-827_ and homologous coronavirus peptides (Supplemental Figure 1, D–F).

**Figure 2:**
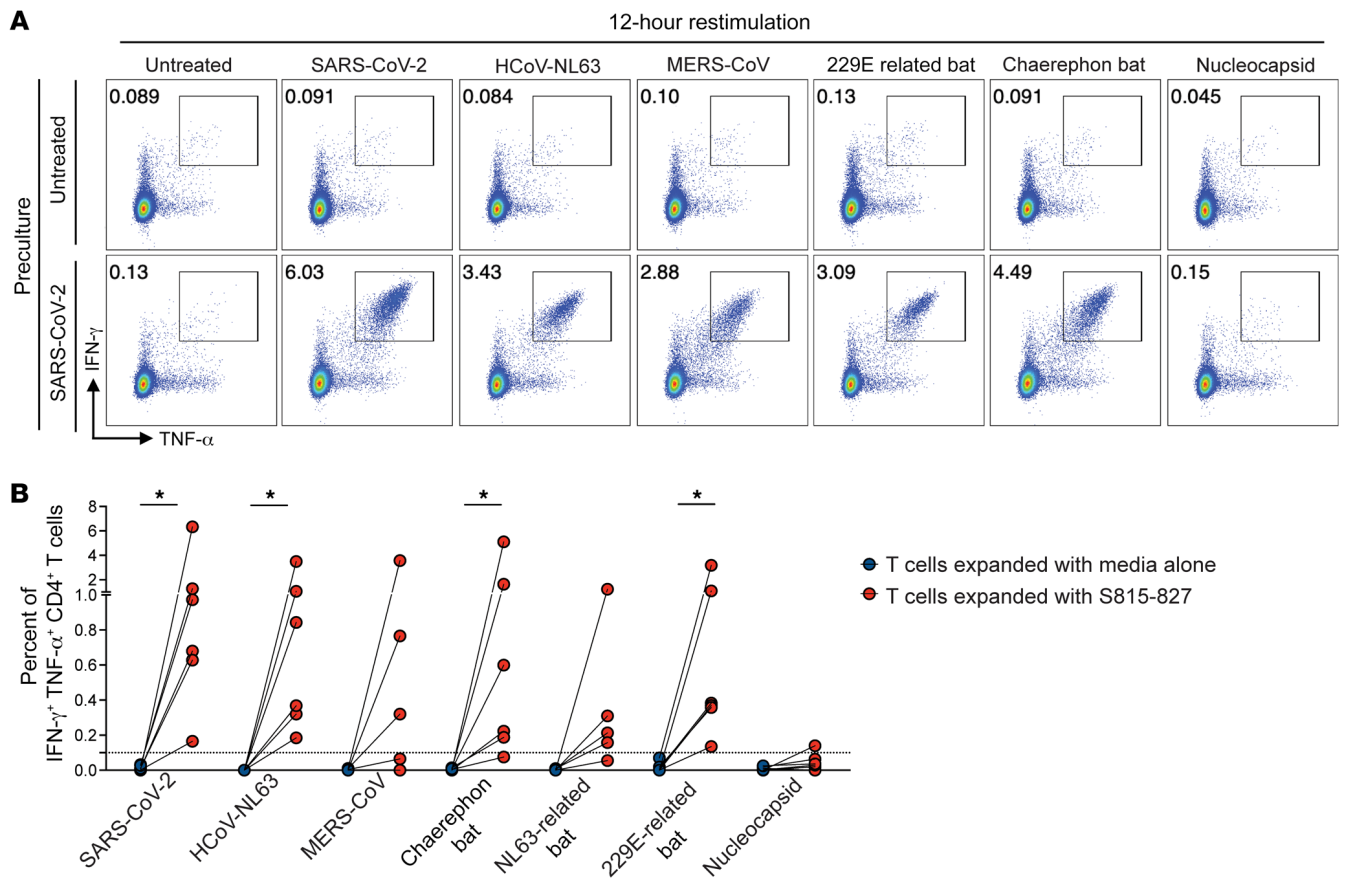
S_815-827_-specific CD4^+^ T cells respond to restimulation with homologous peptides from diverse coronaviruses. T cell lines specific for S_815-827_ were generated by expanding PBMCs for 10 days with S_815-827_. After expansion, cells were restimulated for 12 hours with either the same peptide (S_815-827_) or with homologous peptides from diverse coronaviruses and stained for IFN-γ and TNF-α expression. Cells were restimulated with SARS-CoV-2 nucleocapsid peptide pools as a specificity control. Representative flow plots are shown with peptides used for expansion indicated on the left, and peptides used for restimulation indicated at the top are shown (**A**). IFN-γ^+^ TNF-α^+^ CD4^+^ T cells are gated; percentages are indicated. Responses for all donors tested (*n* = 6) are shown (**B**). Wilcoxon’s matched-pairs signed rank test used for statistical comparisons. *P = 0.0332.

We next asked whether S_815-827_-specific CD4^+^ T cells do in fact cross-recognize homologous epitopes from bat coronaviruses. To assess this, we generated T cell lines specific to S_815-827_ over 10 days. We then restimulated these antigen-specific T cell lines with the same antigen (S_815-827_) or with homologous peptides from bat coronaviruses, and then we measured cytokine production by intracellular cytokine staining and flow cytometry analysis. As expected, S_815-827_-specific CD4^+^ T cells responded robustly to restimulation with the same peptide, with significant increases in the percentage of IFN-γ^+^ TNF-α^+^ cells as compared with cells that were not cultured with S_815-827_ for 10 days. Interestingly, restimulation with peptides from other coronaviruses also resulted in a robust increase in the percentage of IFN-γ^+^ TNF-α^+^ cells over control conditions (Figure 2), suggesting that some S_815-827_-specific T cells were cross-reactive. Overall, S_815-827_-specific CD4^+^ T cells from all responders produced cytokines when stimulated with bat coronaviruses.

To definitively show that vaccinated individuals have true cross-reactive T cells (meaning the same CD4^+^ T cell clonotypes recognizing S_815-827_ and homologous bat coronavirus peptides), we performed the ViraFEST assay. The ViraFEST assay uniquely pairs antigen-specific memory T cell responses and their cognate T cell receptors (TCRs), with the specific antigen stimulating this response after a 10-day T cell culture with relevant antigen followed by TCR Vβ CDR3 sequencing (32). We previously used this assay to identify SARS-CoV-2 and HCoV cross-reactive T cells in COVID-19 convalescent patients (19). Cross reactivity is defined by the functional expansion of the same CD4^+^ TCR clonotypes in response to multiple coronavirus peptides.

**Figure 3:**
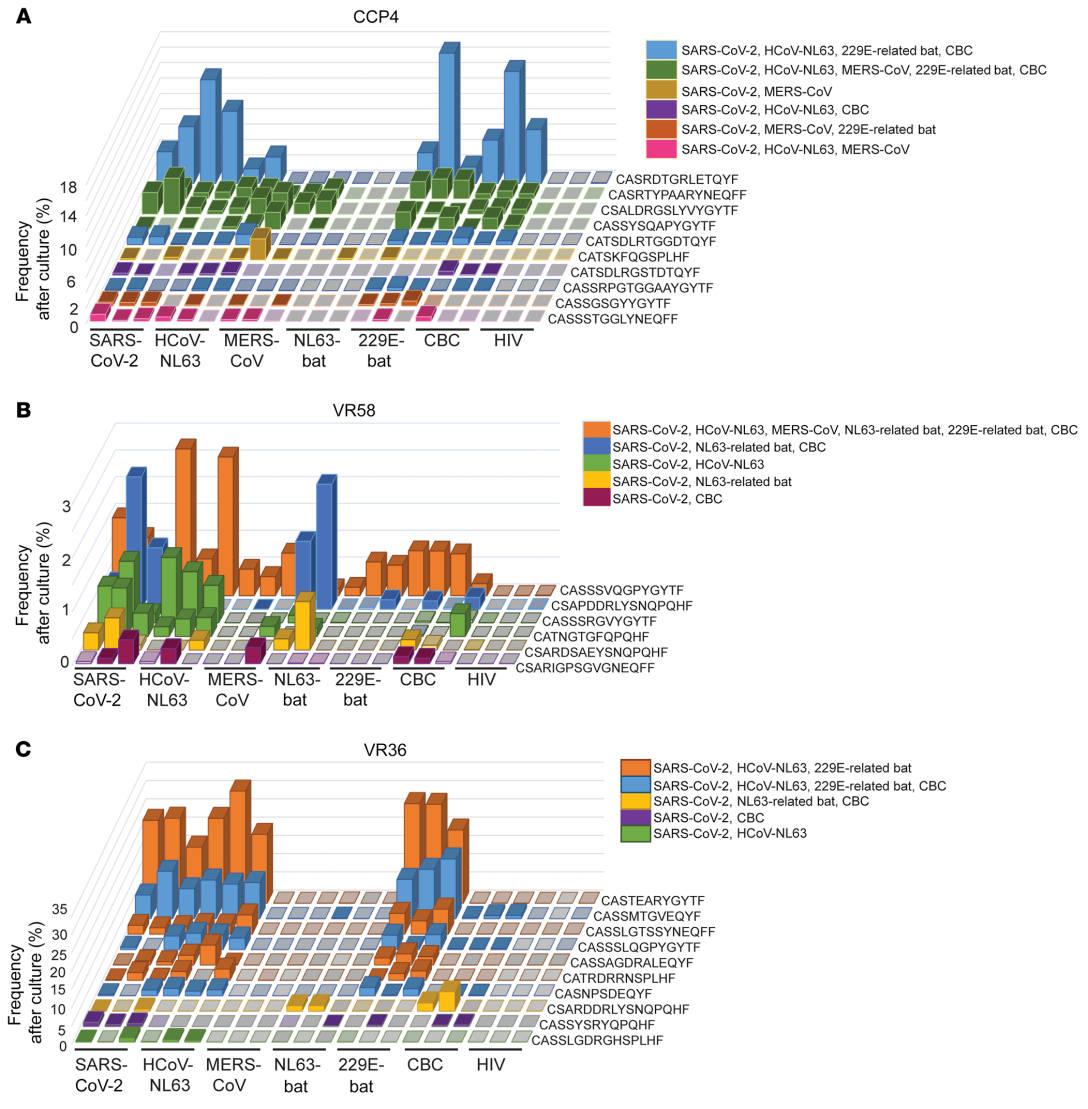
CD4^+^ T cell clonotypes that cross-recognize S_815-827_ and homologous peptides from diverse coronaviruses are present in vaccinated donors. PBMCs isolated from 3 donors (CCP4, VR36, and VR58) were expanded for 10 days with S_815-827_ or homologous peptides from HCoV-NL63, MERS-CoV, NL63-bat, 229E-bat, and CBC. HIV-1 Nef peptides were included as a specificity control. After culture, CD4^+^ T cells were isolated and TCR Vβ CDR3 sequencing was done to identify antigen-specific memory T cells that expanded in response to relevant antigen (ViraFEST assay). Cross-reactivity was defined by the functional expansion of the same CD4^+^ TCR clonotypes in response to multiple coronavirus peptides. Peptide coculture was done in triplicate. Data are shown as the (%) frequency after culture (*y* axis) of antigen-specific CD4^+^ T cell clonotypes (*z* axis) for all peptide pools tested (*x* axis). Solid colors represent significant clonotypic expansion in response to the indicated antigenic peptide pool(s), whereas translucent colors indicate the clonotype was present at low frequency in the well but did not significantly expand. Gray colors indicate the relevant TCR clonotype was not detected in that well. Different colors indicate different patterns of cross-reactive T cells shown in a key above each figure. Cross-reactive clones for CCP4 (**A**), VR58 (**B**), and VR36 (**C**) are shown, with different patterns of cross-reactive T cells color-coordinated. CCP, COVID-19 convalescent patient; VR, vaccine recipient; NL63-bat, NL63-related bat; 229E-bat, 229E-related bat; CBC, Chaerephon bat coronavirus; HIV, HIV-1 Nef.

We performed the ViraFEST assay using PBMCs from 3 donors (CCP4, VR36, and VR58) and peptides from 6 coronaviruses (SARS-CoV2, HCoV-NL63, and MERS-CoV and NL63-related bat, 229E-related bat, and Chaerephon bat coronaviruses) (Figure 3). In all donors tested, we found multiple cross-reactive T cells that recognized S_815-827_ and homologous bat coronavirus peptides (Figure 3 and Supplemental Figure 2). In CCP4, we found TCR clonotypes that recognized the SARS-CoV-2 peptide S_815-827_ and homologous peptides from HCoV-NL63, MERS-CoV, 229E-related bat virus, and Chaerephon bat coronavirus (Figure 3A, indicated in green). Similarly, cross-reactive T cells were observed in VR58 (Figure 3B), such as a TCR clonotype that recognized all 6 coronavirus peptides tested (indicated in orange), and in VR36 (Figure 3C), such as a TCR clonotype that recognized S_815-827_ and peptides from HCoV-NL63, 229E-related bat virus, and Chaerephon bat coronavirus (indicated in blue). CD4^+^ T cell clones specific to NL63-related bat coronavirus peptide were recognized using the ViraFEST assay for VR36 and VR58, despite these donors having a negative result in IFN-γ ELISpot (Figure 1E). This may be because antigen-specific expansion allows for the detection of memory T cell responses that are not picked up by the ELISpot assay.

Since HCoV-HKU1 was recognized by all S_815-827_ responders with the IFN-γ ELISpot assay (Figure 1D), we reasoned that cross-reactive clones identified with ViraFEST might also cross-recognize HCoV-HKU1. To test this, we expanded PBMCs from VR36 and 58 using HCoV-HKU1 peptide and performed the ViraFEST assay. We found that some but not all identified cross-reactive clones recognized HCoV-HKU1 (Supplemental Figure 2). Interestingly, we also found cross-reactive TCRs that did not recognize SARS-CoV2 but recognized other coronaviruses (Supplemental Figure 3), suggesting that a subset of cross-reactive T cells might result from priming by prior HCoV exposure.

## Discussion

Cross-reactive CD4^+^ T cells that can cross-recognize SARS-CoV-2 and endemic HCoVs have been demonstrated in COVID-19–unexposed donors, COVID-19–recovered individuals, and vaccine recipients (19–28). Recent evidence suggests that preexisting HCoV/SARS-CoV-2 cross-reactive T cells in unexposed individuals might lead to better outcomes after COVID-19 infections (33), possibly because cross-reactive memory T cells have faster reactivation and kinetics that allow for robust responses to acute SARS-CoV-2 infection (31).

In this study, we looked at T cell responses to a highly conserved region of SARS-CoV-2 spike (S_815-827_) in COVID-19 mRNA vaccine recipients. S_815-827_ is a highly conserved epitope in alpha and betacoronaviruses (29) and is identical in sequence in coronaviruses closely related to SARS-CoV-2 (Supplemental Table 1). This degree of conservation suggests that S_815-827_ has an important functional role and might be less likely to be affected by escape mutations, making it an appealing target for vaccine strategies.

Our results using IFN-γ ELISpot showed that 40% of vaccinated participants in our cohort mounted T cell responses to S_815-827_, suggesting that a significant percentage of the population might have T cells reactive to this conserved coronavirus epitope. This is consistent with a recent report from Loyal et al. that showed that the 15-mer peptide S_816-830_ is immunodominant and is recognized by most vaccinated individuals (31). In their cohort, Loyal et al. showed that S_816-830_ is targeted by 90% of vaccinated individuals using the activation-induced marker (AIM+) assay, which detects antigen-specific T cell activation regardless of cytokine production and expansive capacity. The AIM+ assay is likely more sensitive than IFN-γ ELISpot, leading to a higher percentage of vaccinated individuals recognizing the conserved epitope in the Loyal et al. study. Furthermore, in our study, we might have underestimated the percentage of S_815-827_ responders because we looked at T cell responses in vaccinees up to 272 days after vaccination, and T cell responses may have waned in this time frame.

Loyal et al. have shown that S_816-830_ is recognized by only 20% of unexposed donors in their cohort versus 50% of COVID-19 convalescent patients and 90% of vaccinated individuals (31), suggesting that in most cases, S_815-827_-reactive T cells are induced or expanded by COVID-19 exposure. This is consistent with the fact that we did not find T cell responses to S_815-827_ with IFN-γ ELISpot in matched prevaccine samples from 3 study participants who had CD4^+^ T cells specific for this epitope after vaccination. However, it is worth noting that our observation might be limited by reduced sensitivity of the IFN-γ ELISpot assay in prevaccine samples for which lower cell numbers were used because of limited cell availability. Furthermore, using the ViraFEST assay, we identified CD4^+^ T cell clonotypes that were cross-reactive to HCoVs and bat coronaviruses but did not recognize SARS-CoV-2 (Supplemental Figure 3), suggesting that a subset of cross-reactive T cells might result from priming by prior HCoV exposure.

In our study, we showed that most S_815-827_ responders also recognized peptides from at least 6 other S_815-827_ homologous coronavirus peptides ex vivo. Furthermore, we showed that S_815-827_-specific T cell lines produced cytokines in response to restimulation with homologous peptides from bat coronaviruses. Finally, we identified truly cross-reactive T cells by identifying CD4^+^ TCR clonotypes that functionally expanded in response to S_815-827_ and homologous bat coronaviruses with the ViraFEST assay. This provides evidence that some vaccinated individuals harbor SARS-CoV-2 and bat coronavirus cross-reactive T cells.

Given the threat posed by future coronavirus pandemics, the development of pan-coronavirus strategies that can enhance protection against potentially zoonotic coronaviruses has garnered increased interest (7). Wang et al. has shown an S2 fusion domain antibody that can cross-neutralize betacoronaviruses, including MERS-CoV in animal models (34). Neutralizing antibodies targeting the S2 fusion domain have also been described in COVID-19 convalescent patients (35) and have been shown to cross-neutralize other betacoronaviruses (36). Additionally, it has been shown that BNT162b2-vaccinated individuals with prior SARS-CoV exposure develop antibodies that can cross-neutralize other sarbecoviruses (37). Collectively, these studies suggest that it might be possible to induce immunity against potentially zoonotic coronaviruses. However, to our knowledge, cross-reactive T cell responses to potentially zoonotic coronaviruses have not yet been studied.

Our results suggest that a large percentage of individuals who received COVID-19 mRNA vaccines have T cells that recognize bat coronavirus peptides, likely due to cross-reactive T cells that target S_815-827_ and homologous bat coronavirus peptides. Additionally, we showed that genetically diverse bat coronaviruses from the beta and alphacoronavirus genus can also be cross-recognized by T cells from vaccinated individuals. Our data support the hypothesis that current COVID-19 vaccinations might enhance protection against certain SARS-CoV-2–related bat coronaviruses. Further, our results provide insight into the development of pan-coronavirus vaccine strategies, such as mRNA vaccines that code for multiple diverse coronavirus peptides, that might have the ability to induce protection against multiple coronaviruses.

## Methods

### Study participants.

COVID-19 convalescent patients were defined as study participants who had tested positive for SARS-CoV-2 by nasal swab PCR test in the past. All the COVID-19 convalescent patients in this study had received 2 doses of mRNA COVID-19 vaccines. The term vaccine recipient refers to participants who had never tested positive for SARS-CoV-2 and had received 2 doses of mRNA COVID-19 vaccines. All study participants worked in health care and/or laboratory settings; 33 participants received the Pfizer-BioNTech (BNT162b2) vaccine, and 5 participants received the Moderna (mRNA-1273) vaccine. Of all participants, 21 were between the ages of 21 and 30 years, 7 were between the ages of 31 and 40 years, 8 were between the ages of 41 and 50 years, and 2 were between the ages of 51 and 60 years. Blood was drawn and processed between June and August 2021. Further details for participants who responded to the S_812-829_ epitope are in Table 2.

### Blood processing.

PBMCs were isolated from whole blood with Ficoll-Paque PLUS gradient centrifugation (GE Healthcare Life Sciences). Briefly, 30 mL blood was overlaid over 15 mL of Ficoll-Paque Plus in a 50 mL conical tube and centrifuged at 400*g* for 25 minutes at 25°C in a swinging-bucket rotor. The PBMC layer was slowly isolated and washed twice with 30 mL wash media (PBS, pH 7.4, 2% heat-inactivated newborn calf serum, 0.1% glucose, 20 U/mL penicillin, 20 μg/mL streptomycin, 12 mM HEPES, pH 7.4) at 450*g* for 10 minutes at 4°C in a swinging-bucket rotor. Cells were then counted and resuspended with the appropriate culture medium relevant for the appropriate downstream application.

For experiments requiring CD8^+^ T cell depletion, Miltenyi Biotec CD8^+^ T Cell Positive Selection kits (130-045-201) were used per the manufacturer’s instructions. All experiments, with the exception of prevaccine samples, were performed using freshly isolated PBMCs.

IFN-γ ELISpot using prevaccine samples was performed using cryopreserved samples. Briefly, cells were frozen in freezing medium (10% DMSO, Sigma-Aldrich D2650; 90% FBS, Gibco 16140-071) at 10–20 million cells/mL in a cryofreezing container at –80°C. Only cells with greater than 90% viability were frozen. Cells were quickly thawed (1–2 minutes) at 37°C and slowly transferred into R50 medium (50% RPMI, Gibco, 61870-036; 50% FBS, Gibco, 16140-071). Before any experiments, cells were thawed and rested for 24 hours at 2 million cells/mL in R10 media (RPMI + 10% FBS + 1% penicillin-streptomycin, Gibco, 15140-122) in a 37°C incubator.

### Serology.

All study participants were tested for antibodies against HCoV-NL63, HCoV-OC43, HCoV-229E, and HCoV-HKU1. Antibody responses were evaluated using ELISA kits purchased from Alpha Diagnostics International (RV-406130, RV-406115, RV-406100, RV-406145) following the manufacturer’s instructions. Briefly, plasma samples were diluted 1:500 and incubated on precoated plates for 1 hour. After appropriate washes, plates were incubated with anti-human IgG HRP conjugates followed by substrate reactions. Plasma samples were isolated from May to August 2021 when T cell analysis was done in this study. The presence of IgG antibody was determined relative to the anti-SARS calibrator provided by the manufacturer, and a threshold index was calculated.

### Peptides and ELISpot assays.

Coronavirus peptide sequences were provided to GenScript and peptides were synthesized by the vendor (95% purity). Upon arrival, peptides were reconstituted with DMSO at a stock concentration of 10 mg/mL. Anti-CD3 antibody (Mabtech, 1 μg/mL) was used as a positive control for each study participant. IFN-γ ELISpot assays were performed as previously described (24). Briefly, ELISpot Pro and ELISpot Plus kits with precoated plates were purchased from Mabtech. The wells were plated with CD8^+^ T cell–depleted PBMCs at 250,000 cells/well for postvaccine samples or 100,000 PBMCs/well for prevaccine samples. Cells were cultured with 1 μg/mL of each peptide in R10 media for 20 hours at 37°C, and then processed according to the manufacturer’s protocol. ELISpot plates were read by a blinded independent investigator on an AID iSpot Spectrum using vendor-provided software that reported SFU/well. Spot/million cells was calculated by multiplying spots/well by the appropriate dilution factor. Stimulation index (fold-change over untreated controls) for each donor was calculated by dividing the SFUs of peptide condition by the SFUs of the untreated control. Four replicates were run for each condition, and the replicate furthest from the median was not used. The mean of replicate values was used for plotting. A positive response was defined as a mean SFU of greater than or equal to 20 and a mean SI of greater than or equal to 3.

### T cell expansion culture assay.

First, 10–20 million PBMCs were cultured in R10 with 10 U/mL IL-2 and 5 μg/mL S_815-827_ peptide for 10–12 days as previously described (24). The media were not changed during this period. The cells were then washed and replated in fresh R10 with 10 U/mL IL-2 and rested 1 day in a 37°C incubator before they were stimulated again with 1 μg/mL peptide with protein transport inhibitors (GolgiPlug, BD Biosciences, 555029, 1 μg/mL; GolgiStop, BD Biosciences, 554724, 0.7 μg/mL) and antibodies against CD28 (BD Biosciences, 555725) and CD49d (BD Biosciences, 555501). Restimulation was done with either the same peptide (S_815-827_), homologous peptide pools listed in Table 1, or a SARS-CoV-2 nucleocapsid peptide pool (12 mer, 13 mer, or 17 mer peptides with 10 amino acid overlaps made up of 59 peptides total, ordered from BEI Resources and reconstituted in DMSO at 10 mg/mL as previously described; ref. 24). After a 12-hour incubation, the cells were washed and stained with annexin V (BV-421, BD Biosciences, 563973) and antibodies against CD3 (APC-Cy-7, BioLegend, 300426), CD4 (PerCP-CY-5.5, BioLegend, 300530), and CD8 (BV-605, BioLegend, 301040). The cells were then fixed, permeabilized, and stained intracellularly for TNF-α (PE-Cy-7, BD Biosciences, 557647) and IFN-γ (APC, BD Biosciences, 506510). Flow cytometry was performed on a BD FACS LSRFortessa flow cytometer, and data were analyzed using FlowJo software, version 10. Data on a minimum of 100,000 events in the lymphocyte gate were collected and analyzed.

### Identification of epitope-specific T cells.

Coronavirus peptides from SARS-CoV2, HCoV-NL63, HCoV-HKU1, and MERS-CoV and NL63-related bat, 229E-related bat, and Chaerephon bat coronaviruses (listed in Figure 1) were used to stimulate CD4^+^ T cells in the ViraFEST assay as described previously (18). Briefly, 2 × 10^6^ PBMCs were plated in culture medium (IMDM, 5% human AB serum, 10 IU/mL IL-2, 50 μg/mL gentamicin) with 1 μg/mL of each peptide, a negative control HIV-1 Nef peptide pool (NIH AIDS Reagent Program ARP-12545), or without peptide. Each assay condition was performed in triplicate. On days 3 and 7, half the media was removed and replaced with fresh culture media. On day 10, cells were harvested and CD4^+^ T cells were isolated using the EasySep CD4+ T cell isolation kit (STEMCELL, 17952). DNA was extracted from cultured CD4^+^ T cells using the QIAmp Micro-DNA kit according to the manufacturer’s instructions (QIAGEN). TCR-Seq of DNA extracted from cultured CD4^+^ T cells was performed by the Johns Hopkins FEST and TCR Immunogenomics Core Facility (FTIC) using the Oncomine TCR Beta Short-Read Assay (Illumina Inc.). Samples were pooled and sequenced on an Illumina iSeq 100 using unique dual indexes. Data preprocessing was performed to eliminate nonproductive TCR sequences and to align and trim the nucleotide sequences to obtain only the CDR3 region. Sequences not beginning with C or ending with F or W and having fewer than 7 amino acids in the CDR3 were eliminated. Resultant processed data files were uploaded to our publicly available MANAFEST analysis web application (http://www.stat-apps.onc.jhmi.edu/FEST/) to bioinformatically identify antigen-specific T cell clonotypes. Clones were considered positive based on the following criteria: (a) significantly expanded in the culture of interest (in 2 of 3 replicate wells) compared with the reference culture (PBMCs cultured with 10 IU/mL IL-2 and HIV-1 Nef pool or media without peptide) at an FDR less than the specified threshold (<0.05; default value); (b) having an odds ratio greater than 5 (default value); and (c) having a minimum of 0.1% frequency in 2 of 3 replicate wells. To identify cross-reactive responses, we used statistical criteria established previously (38).

### HLA haplotyping.

High-resolution class II typing was performed by the Johns Hopkins Hospital Immunogenetics Laboratory. HLA-binding predictions were made using the Immune Epitope Database (IEDB) (http://www.iedb.org) between December 8, 2021 and December 9, 2021. The IEDB-recommended 2.22 prediction method (which uses the consensus approach, combining NN-align, SMM-align, CombLib, and Sturniolo if any corresponding predictor is available for the molecule and otherwise uses NetMHCIIpan) was used to make predictions (39, 40). The 13-mer peptide sequence RSFIEDLLFNKVT was used to make binding predictions, and the MHC II alleles with percentile rank less than 10 (lower percentile rank indicates higher affinity) were selected and listed in order of increasing percentile rank in Table 2.

### Statistics.

All statistical analyses were performed using GraphPad Prism 9.2.0. Comparisons between 2 groups were done with a Mann-Whitney test (if unpaired) and Wilcoxon’s matched-pairs signed rank test (if paired). Comparisons between multiple groups were done using Friedman’s test with Dunn’s multiple-comparison test. A *P* value of less than 0.05 was considered statistically significant.

### Study approval.

The study was approved by the IRB of Johns Hopkins University. Written informed consent was obtained from all participants prior to their inclusion in the study.

## Author contributions

BAW, AGD, and CCG performed experiments and analyzed data. SC analyzed data. KNS and JNB supervised experiments. BAW wrote the manuscript. AGD, CCG, SC, KNS, and JNB edited the manuscript.

## Supplementary Material

Supplemental data

## Figures and Tables

**Table 2:**
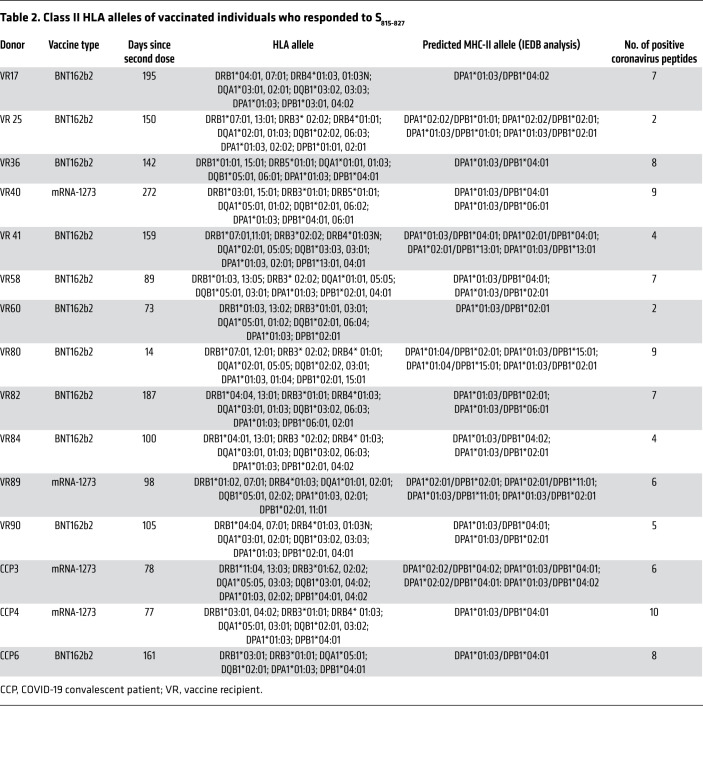
Class II HLA alleles of vaccinated individuals who responded to S_815-827_

**Table 1:**
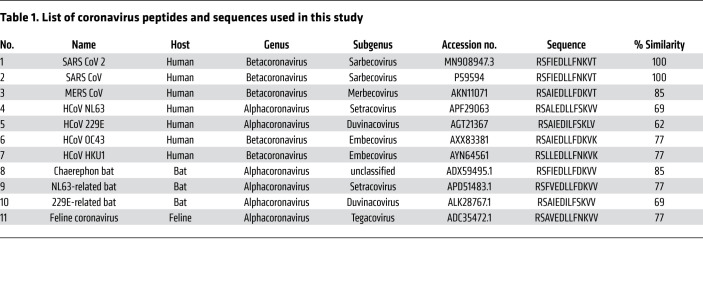
List of coronavirus peptides and sequences used in this study

## Version 1 01/21/2022

In-Press Preview

## Version 2 03/01/2022

Electronic publication
