# Supplementary material for: CD4+ T cells from COVID-19 mRNA vaccine recipients recognize a conserved epitope present in diverse coronaviruses
Source: J Clin Invest. 2022 Mar 1;132(5):e156083. doi: 10.1172/JCI156083 (PMC8884904; doi:10.1172/JCI156083)
Supplement: Supplemental data [file jci-132-156083-s058.pdf]

Supplemental Figure 1

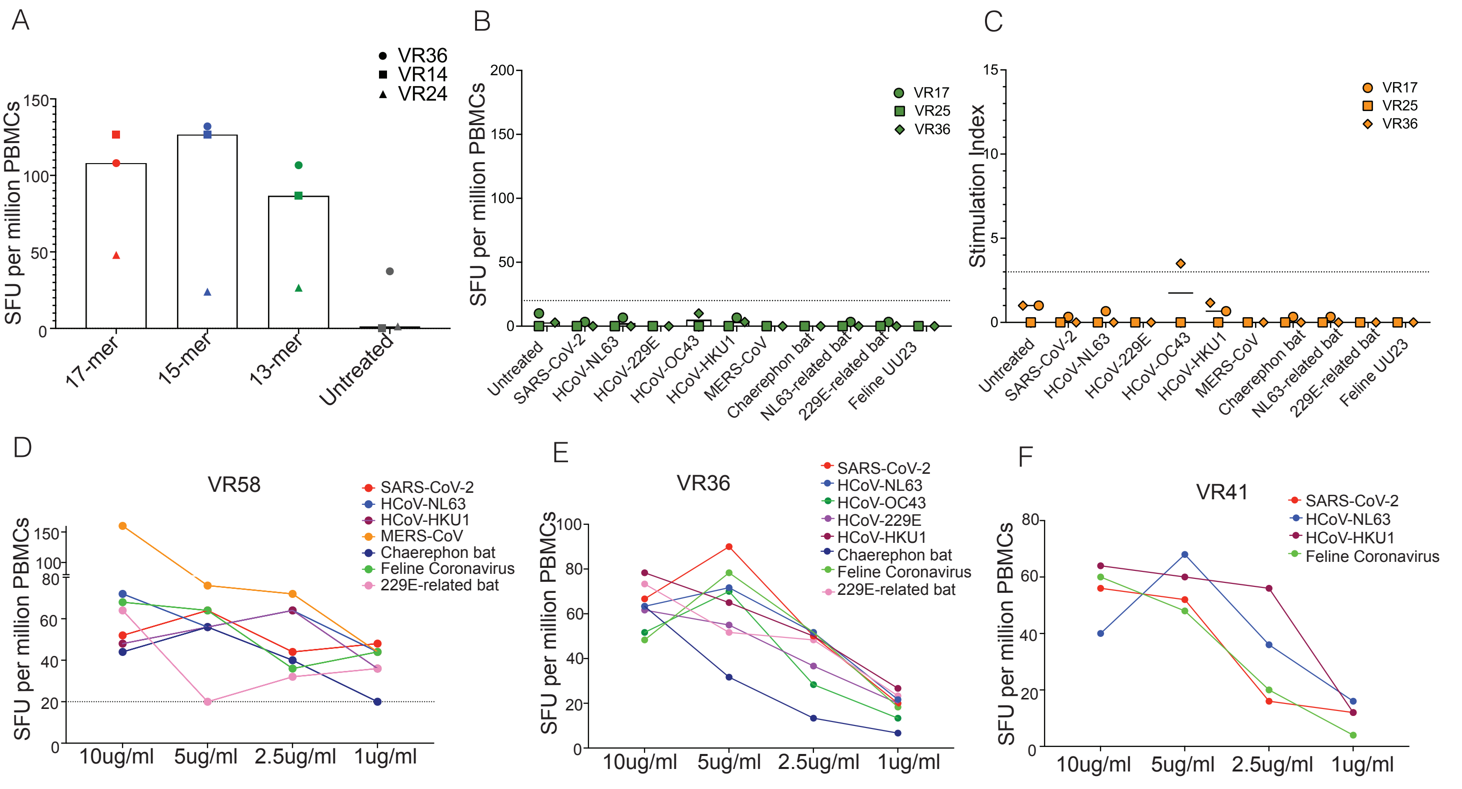

Supplementary Figure 1: S815-827 reactive T cells are not detected in pre-vaccination samples, and have similar functional avidity to T cells reactive to homologous peptides. The 13-mer peptide was determined by isolating PBMCs from 3 donors previously shown to recognize a 17-mer peptide S813-829 (14). 15-mer and 13-mer truncated peptides were synthesized and an IFN-γ ELISpot assay was performed in triplicates to determine the minimal peptide recognized (A). Responses to S813-829 and homologous coronavirus peptides prior to vaccination were assessed in 3 donors (B-C). Briefly, cryopreserved PBMCs isolated prior to COVID-19 vaccinations were thawed and IFN-γ ELISpot assay was performed with indicated peptides. Lines indicate SFU=20 (B) and SI=3 (C). CD4+ T cell avidity to coronavirus peptides was tested by titrating peptide concentration in the IFN-γ ELISpot assay (D-F). Briefly, CD8+ T cell depleted PBMCs were isolated from 3 vaccinated individuals, and an IFN-γ ELISpot assay was done in triplicate with S815-827 or homologous peptides titrated serially to determine T cell avidity. Mean of replicates was used to plot spot forming units (SFU) (D-F).

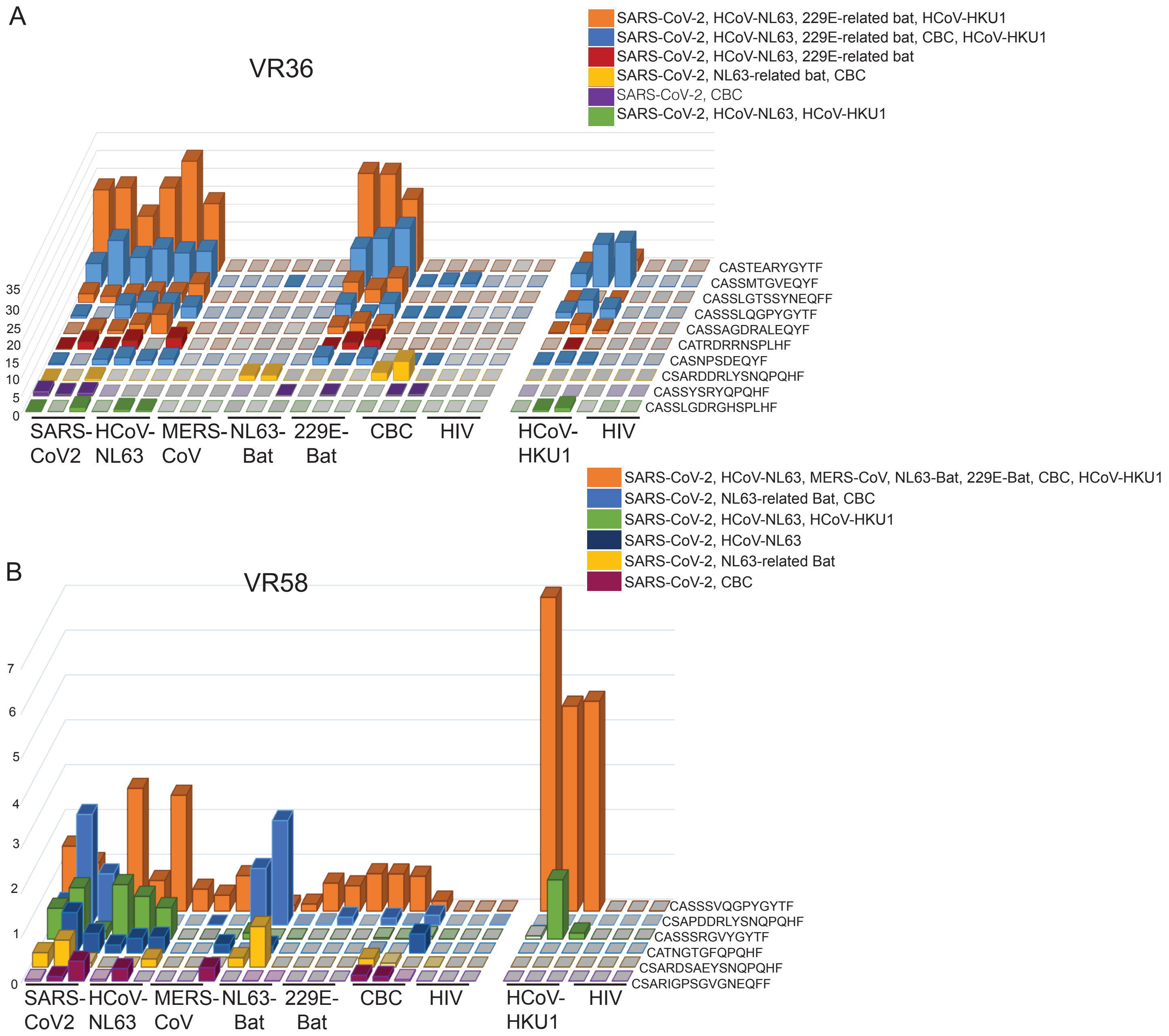

Supplemental Figure 2: Cross-reactive CD4+ T cell clonotypes that react to diverse coronaviruses are found in vaccinated donors. PBMCs were expanded for 10 days with S815-827 or homologous peptides from HCoV-NL63, MERS-CoV, NL63-related bat, 229E-related bat, and Chaerephon bat coronavirus (CBC) (shown on Figure 3). On separate time point, PBMCs were expanded with HCoV-HKU1. HIV-1 Nef peptides were included as a specificity control at both time points. Following culture, CD4+ T cells were isolated and TCR Vβ CDR3 sequencing was done to identify antigen-specific memory T cells that expanded in response to relevant antigen (Vira-FEST assay). Cross-reactivity was defined by the functional expansion of the same CD4+ TCR clonotypes in response to multiple coronavirus peptides. Peptide co-culture was done in triplicate. Data are shown as the (%) frequency after culture (y axis) of antigen-specific CD4+ T cell clonotypes (z axis) for all peptide pools tested (x axis). Solid colors represent significant clonotypic expansion in response to the indicated antigenic peptide pool(s), whereas translucent color indicates the clonotype was present at low frequency in the well, but did not significantly expand. Gray indicates the relevant TCR clonotype was not detected in that well. Cross-reactive clones for VR36 (A), and VR58 (B) are shown, with different patterns of cross-reactive T cells color coordinated. NL63-Bat = NL63-related Bat, 229E-Bat = 229E-related Bat, CBC = Chaerephon bat coronavirus, HIV = HIV-1 Nef

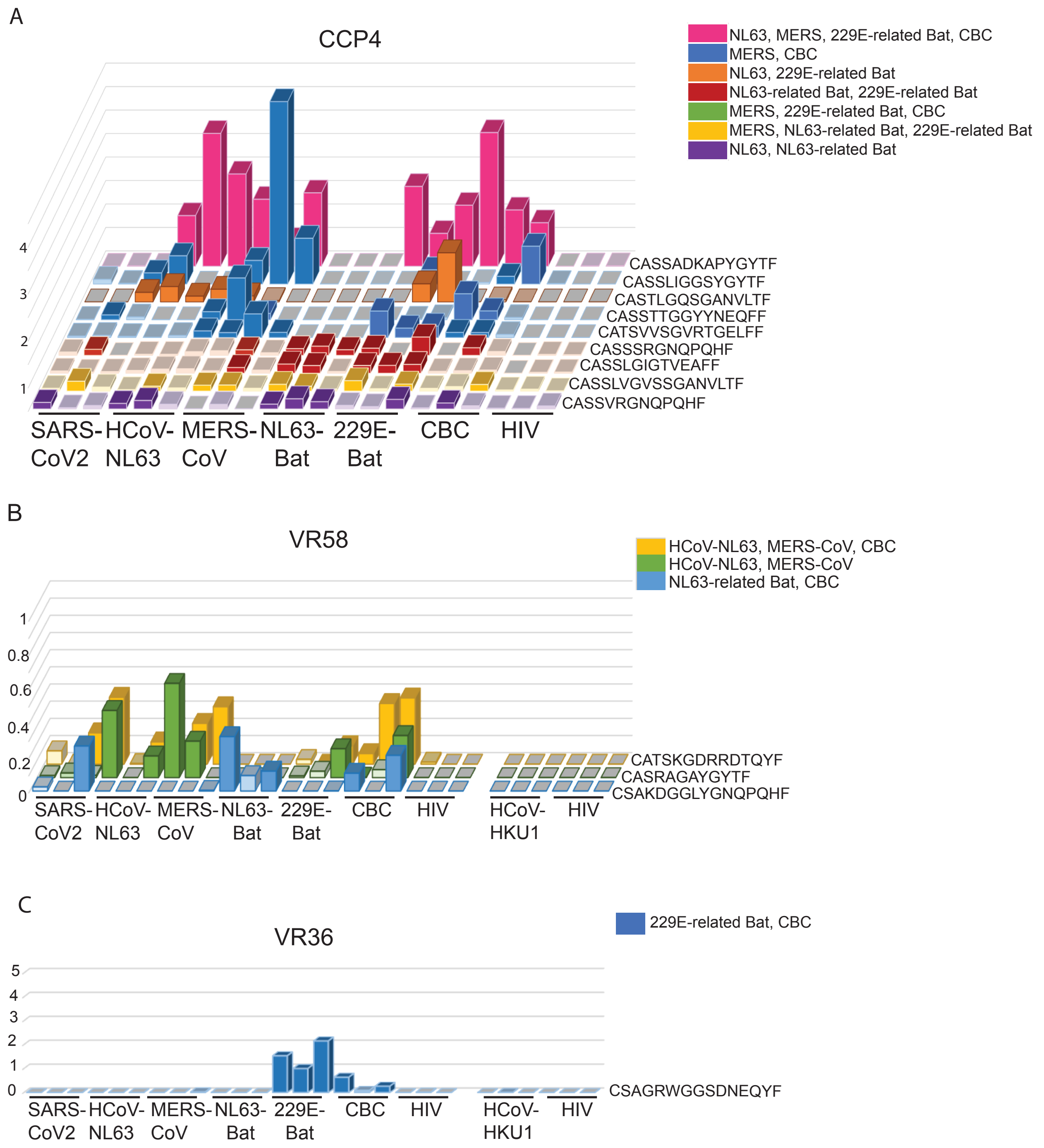

Supplemental Figure 3: Cross-reactive CD4+ T cell clonotypes that react to diverse coronaviruses (excluding S815-827) are found in vaccinated donors. PBMCs were expanded for 10 days with S815-827 or homologous peptides from HCoV-NL63, MERS-CoV, NL63-related bat, 229E-related bat, and Chaerephon bat coronavirus (CBC). At a separate time point, PBMCs from VR36 and 58 were expanded with HCoV-HKU1. HIV-1 Nef peptides were included as a specificity control at both time points. Following culture, CD4+ T cells were isolated and TCR Vβ CDR3 sequencing was done to identify antigen-specific memory T cells that expanded in response to relevant antigen (Vira-FEST assay). Cross-reactivity was defined by the functional expansion of the same CD4+ TCR clonotypes in response to multiple coronavirus peptides. Peptide co-culture was done in triplicate. Data are shown as the frequency (%) after culture (y axis) of antigen-specific CD4+ T cell clonotypes (z axis) for all peptide pools tested (x axis). Solid bars represent significant clonotypic expansion in response to the indicated antigenic peptide pool(s), whereas translucent color indicates the clonotype was present at low frequency in the well, but did not significantly expand. Gray indicates the relevant TCR clonotype was not detected in that well. Colors indicate different patterns of cross-reactive T cells. Cross-reactive clones for CCP4 (A), VR58 (B), and VR36 (C) are shown, with different patterns of cross-reactive T cells color coordinated.

Supplemental Figure 1: S815-827 is identical in sequence between SARS-CoV-2 and SARS-related coronaviruses in the subgenus sarbecovirus and genus betacoronavirus listed.

Virus Name	Host species	Genus	Subgenus	Sequence	Residue	Accession	Source DOI
SARSCoV 2	Human	Betacoronavirus	Sarbecovirus	RSFIEDLLFNKVT	815-827	MN908947.3	10.1038/s41586 -020-2008-3
Longquan_140	Rhinolophus_monoceros	Betacoronavirus	Sarbecovirus	RSFIEDLLFNKVT	784-796	KF294457	10.1016/j.virol.2017.03.019
RaTG13	Rhinolophus_affinis	Betacoronavirus	Sarbecovirus	RSFIEDLLFNKVT	811-823	MN996532	10.1038/s41586 -020-2012-7
CoVZC45	Rhinolophus_sinicus	Betacoronavirus	Sarbecovirus	RSFIEDLLFNKVT	788-800	MG772933	10.1038/s41426 -018-0155-5
CoVZXC21	Rhinolophus_sinicus	Betacoronavirus	Sarbecovirus	RSFIEDLLFNKVT	787-799	MG772934	10.1038/s41426 -018-0155-5
GX-P4L	Manis_javanica	Betacoronavirus	Sarbecovirus	RSFIEDLLFNKVT	809-821	MT040333	10.1038/s41586 -020-2169-0
RacCS203	Rhinolophus_acuminatus	Betacoronavirus	Sarbecovirus	RSFIEDLLFNKVT	787-799	MW251308	10.1038/s41467 -021-21240-1
Rc-o319	Rhinolophus_cornutus	Betacoronavirus	Sarbecovirus	RSFIEDLLFNKVT	777-789	LC556375	10.3201/eid2612.203386
RpYN06	Rhinolophus_pusillus	Betacoronavirus	Sarbecovirus	RSFIEDLLFNKVT	788-800	MZ081381	10.1016/j.cell.2021.06.008
PrC31	Rhinolophus_spp	Betacoronavirus	Sarbecovirus	RSFIEDLLFNKVT	788-800	MW703458	10.1080/22221751.2021.1964925
